# Supplementary material for: Association between oral microbiome and five types of respiratory infections: a two-sample Mendelian randomization study in east Asian population
Source: Front Microbiol. 2024 Apr 10;15:1392473. doi: 10.3389/fmicb.2024.1392473 (PMC11039966; doi:10.3389/fmicb.2024.1392473)
Supplement: Supplementary file 1 [file Data_Sheet_1.zip › Supplementary Materials/MR plots of saliva/Tonsillitis/s__Streptococcus_pseudopneumoniae_O_mgs_2474/funnel.pdf]

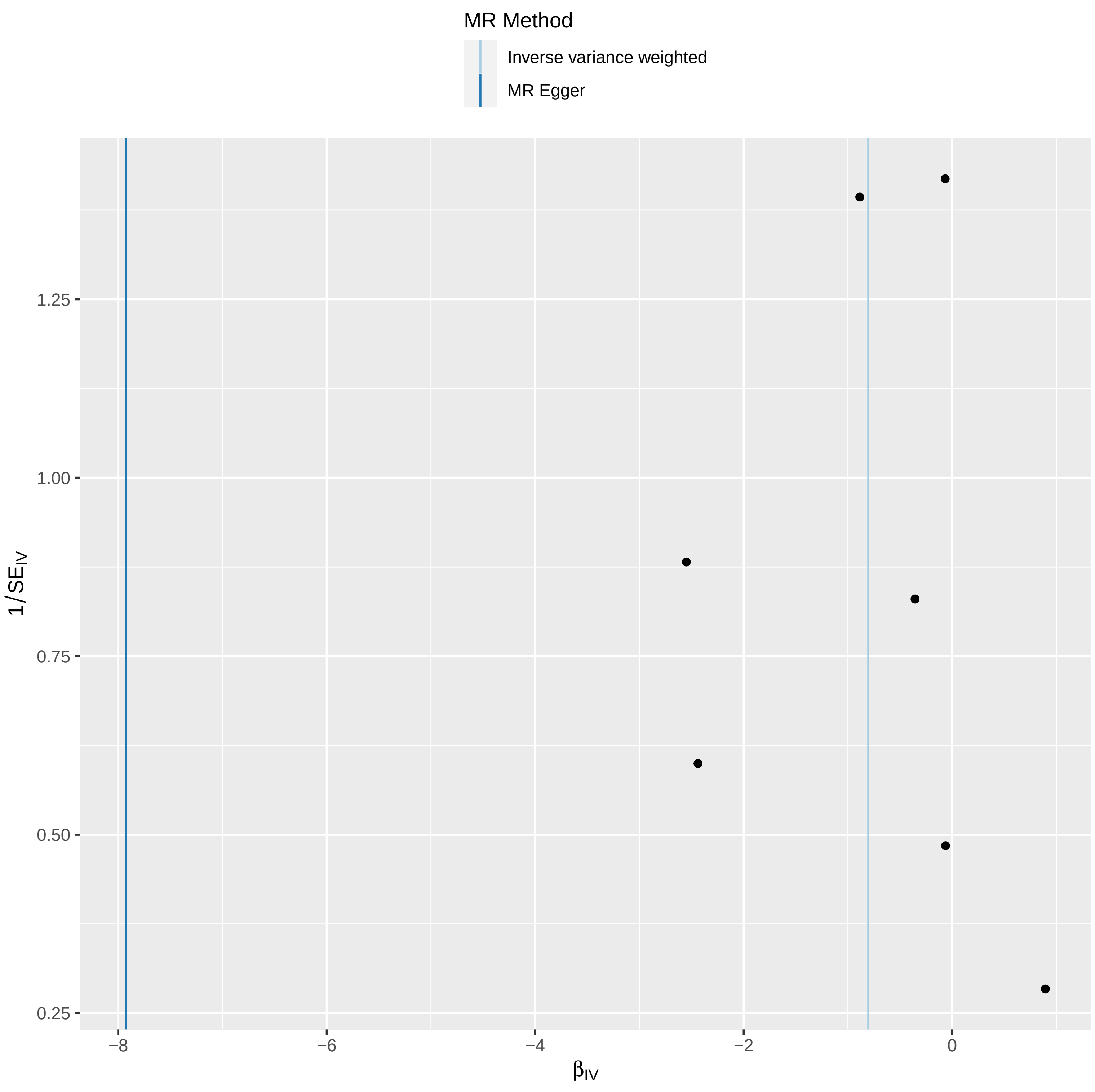
MR Method
Inverse variance weighted
MR Egger
1/SE_IV
1.25
1.00
0.75
0.50
0.25
−8
−6
−4
−2
0
β_IV
